# Evaluation of the efficacy of a simulation model used in oral and maxillofacial surgery education

**DOI:** 10.1186/s12909-024-05307-3

**Published:** 2024-03-19

**Authors:** Aysegul Erten Taysi, Nuri Mert Taysi, Soner Sismanoglu

**Affiliations:** 1 Faculty of Dentistry, Department of Oral and Maxillofacial Surgery, Altinbas University, Istanbul, 34147 Turkey; 2 Faculty of Dentistry, Department of Oral and Maxillofacial Surgery, Istanbul University-Cerrahpasa, Istanbul, 34098 Turkey; 3 Faculty of Dentistry, Department of Restorative Dentistry, Istanbul University-Cerrahpasa, Istanbul, 34098 Turkey

**Keywords:** Dental education, Mannequin, Tooth extraction, Anxiety, Self-confidence

## Abstract

**Background:**

The traditional teaching methods of dental education are gradually being replaced with futuristic education methods based on the usage of educational tools such as mannequin-based simulation models and virtual reality. However, the effectiveness of mannequin-based simulation models as a learning method in the field of oral surgery remains unclear. This study aims to investigate the efficacy of training on a tooth extraction model (TEM) in view of undergraduate dental students’ experience and perception of their education.

**Methods:**

A quasi-experimental trial was implemented with two consecutive year classes, totaling 136 students at the Dentistry Faculty of Altinbas University, Turkiye. Two cohorts were created from dental students in the classes of 2023 and 2022 graduates. Cohort 1 (*n* = 71) received 14 h of theoretical education followed by 10 h of preclinical education on TEM. Cohort 2 (*n* = 65) received only 14 h of theoretical education. An anonymous questionnaire was prepared with four main sections including the preferences of learning style, participants’ perceptions of the preclinical training methods, the students’ competency and free text comments. Students’ opinions were quantified with both 7-point Likert scales and thematic analysis. Anxiety levels were measured with the interval scale of anxiety response (ISAR). Descriptive statistics, inferential statistical and thematic analyses were conducted according to survey responses. Student characteristics were summarized and compared for two cohorts using a t-test. For all statistical analyses, the significance level was set at*P ≤ 0.05.*

**Result:**

Cohort 1 was more comfortable with sequential motions performed with the forceps (*P* = 0.033) and felt more ready for their first clinical tooth extraction experience (*P* = 0.028). Cohort 2 showed a significantly higher preference for textbooks among supplementary materials (*P* = 0.04); however, they tended to exhibit lower self-confidence and higher anxiety levels, though without any statistical significance (*P* > 0.05).

**Conclusion:**

It is clear that the students who have yet to start seeing patients benefit from increased practice with training models, which adequately reflect and represent real-life situations encountered in everyday practice.

**Supplementary Information:**

The online version contains supplementary material available at 10.1186/s12909-024-05307-3.

## Introduction

Considering the inevitable changes brought on by technological breakthroughs, training for real-life experiences with simulations has been playing an increasingly important role in modern education methods. Healthcare education is no exception to this trend [1]. Simulation training is a favored approach in dental education from the onset of preclinical education and can take the form of either mannequin-based simulation education or virtual reality-based simulation, though the latter is not easily accessible to every university due to economic disparities [2–4]. For this reason, several prior studies have focused only on improving course quality by mannequin-based simulation education.

Investigations on dental students throughout the world have shown a considerable difference in both duration and content of the dental curriculum with regards to teaching of local anesthesia techniques, tooth color determination systems, root canal treatment and fixed prosthesis [5–10]. In Turkiye, dental education takes five years, and undergraduate students take the Introduction to Oral and Maxillofacial Surgery course in the fall and/or spring semester of their third year, depending on the curriculum of diverse dentistry faculties. The theoretical content of these courses is the same for all schools, according to the Turkiye Higher Education Quality Council, but there are differences in the practical aspects of oral surgery education. Although some departments combine the theoretical course with preclinical training on mannequin models, most aim to hone students’ skills by having them perform tooth extraction directly on patients following a clinical observation [11, 12]. Throughout the common theoretical curricula, undergraduate students in the fall term of the fourth year would be expected as novice clinicians to be able to perform tooth extraction on their patients.

The use of mannequin-based simulation models in preclinical education has been valued by educators in fields such as prosthodontics and restorative dentistry [9, 10, 13]. Despite many dental schools worldwide have used mannequins for more than 15 years in those fields, their specifications and the effects of their use remain unclear. Unsurprisingly, given the use of mannequin-based simulation models in the oral surgery fields in the undergraduate program is relatively newer and trendier than that of other fields, there is scarce investigation and no exact information about the effectiveness of training of oral surgery procedures like forceps extraction on those kind of models [7, 9, 14–16]. In addition, various studies indicate that tooth extraction has been known as one of the specific issues of oral surgery field in which undergraduate dental students and trainees feel unprepared, stressed, and technically insecure [7, 15]. At this point, it is clear that investigations in teaching tooth extraction methods may result in increasing graduated dentists’ preparedness to perform extraction in independent clinical practice, resulting in better patient care with dentists who have higher levels of self-confidence in their abilities [7, 10].

This study contributes data from undergraduate students in oral surgery clinics to partially fill this void. Its purposes are threefold. The first is to explore whether students feel more comfortable in performing tooth extraction following training on a tooth extraction model (TEM). The second purpose is to compare the anxiety and self-confidence levels of dental students who have used TEM in their preclinical education with those of students who have not. The last purpose is to understand dental students’ opinion on oral and maxillofacial surgery lectures.

## Materials and methods

### Study design

This study was carried out with the approval of the Ethics Committee of Altinbas University in accordance with the Declaration of Helsinki (registration/file number: 117/28,714). The design was a single center, a parallel group, quasi-experimental trial and involved the graduating classes of 2023 (71 students) and 2022 (65 students).

### Inclusion and exclusion criteria for participants

The target population was dental students as novice clinicians performing tooth extraction at integrated clinics of the Altinbas University hospital. Eligible participants were all students in the relevant graduating classes who have performed their first tooth extraction on a live patient. Exclusion criteria was set to be the students who have not performed tooth extraction on a live patient. All students from the both classes fulfilled the inclusion criteria (a total of 136 students).

### Interventions

As the study employed a quasi-experimental cohort design, Cohort 1 and Cohort 2 were designed to be composed of undergraduate dental students who were part of graduating classes of 2023 (71 students) and 2022 (65 students), respectively. Cohort 1 (*n* = 71) received 14 h of theoretical education (2 of 14 h were video lectures) followed by 10 total hours of face-to-face preclinical education on TEM. Cohort 2 (*n* = 65) received only 14 h of theoretical education (2 of 14 h were video lectures) without any preclinical experience on TEM. Both group received the same theoretical curriculum via online classes by the same lecturer (A.E.T), who was solely responsible for the educational interventions used in the study and has been teaching in the university setting in the oral and maxillosurgery field for the past 6 years.

### Forming the study groups

Before the second half of the 2019-20 academic year, the World Health Organization (WHO) declared COVID-19 a pandemic; subsequently, our institution made the decision to suspend all in-person clinical and preclinical education in the spring term in adherence to the announcement of Turkiye’s Council of Higher Education that online education should be prioritized due to the pandemic. The oral and maxillofacial surgeon (A.E.T) promptly adapted to teach theoretical aspects of tooth extraction through distance education using digital platforms.

In the following academic year (2020–2021), which was dubbed a normalization period after COVID-19, our institution carried on with theoretical education via distance learning, while conducting both preclinical trainings and dental internships onsite within the limitations of social distancing. In this period, a total of 71 undergraduate students in the third year of dentistry education were designated as Cohort 1 and had a chance of to train on TEMs in addition to taking theoretical courses prior to their dental internship program. Meanwhile, a total of 65 undergraduate students enrolling in the fourth year of dentistry education, who were unfortunately equipped only with theoretical education received during the pandemic, and who began their dental internship program to gain firsthand experience in providing dental care to patients, were designed as Cohort 2. Whereas Cohort 1 was given 14 h of theoretical education (2 of 14 h were video lectures) and 10 total hours of preclinical education on TEM, Cohort 2 received only 14 h of theoretical education (2 of 14 h were video lectures) without any preclinical experience on TEM.

### Educational materials and environment

**Fig. 1:**
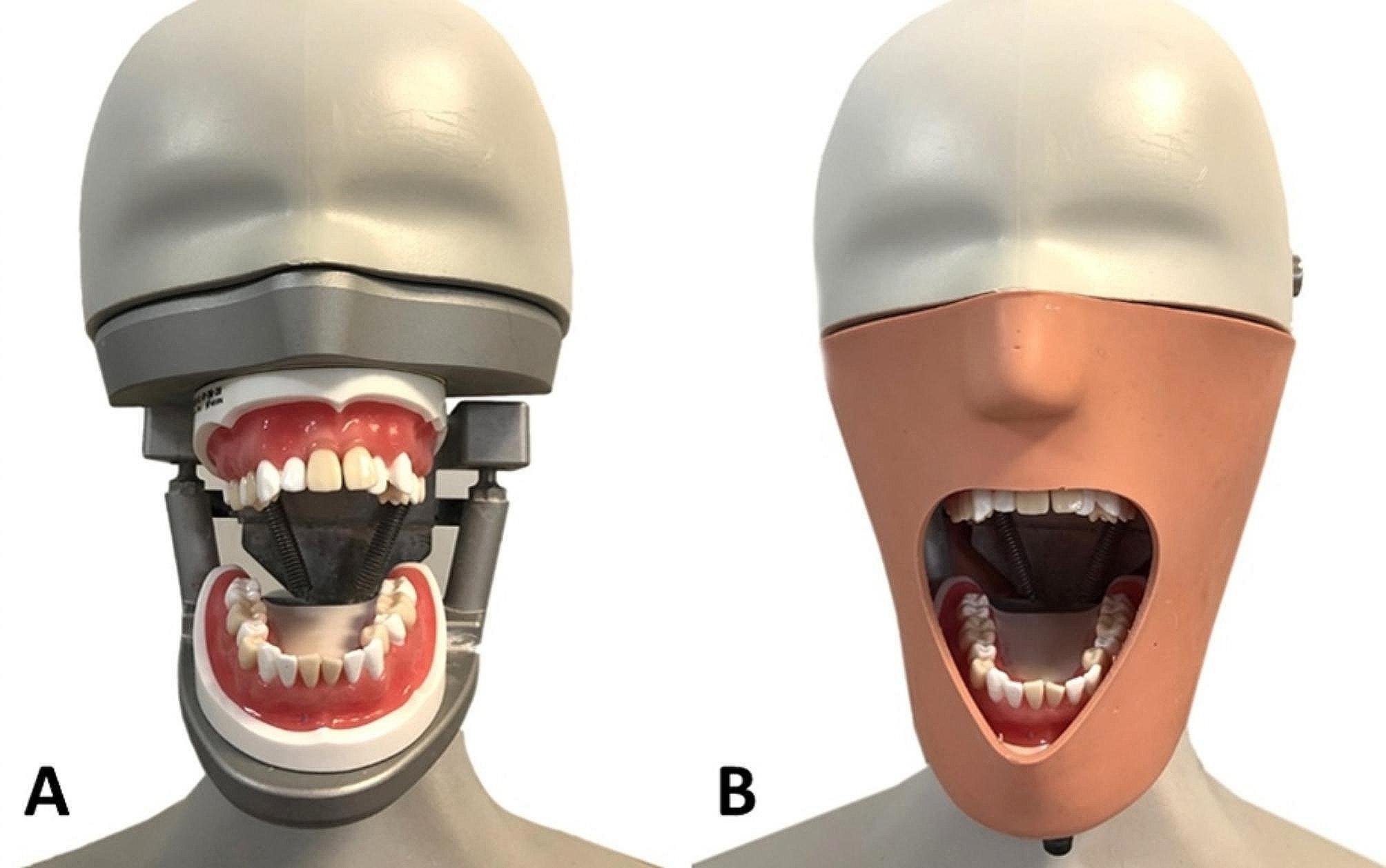
The TEM jaw was fixed to vertical adjustment in the bottom and upper plates of a mannequin head (**A**) with a face mask (**B**)

For theoretical education, one of the authors (A.E.T.), as an assistant professor, exhibited commitment to the current curriculum. Online classes were done over the internet via the distant learning platform of the university. For preclinical education, TEMs (SUG2004-UL-SP-DM-28; Nissin Dental Products Inc., Kyoto, JAPAN) were used as a specific educational material for preclinical exercises for this trial. A TEM is composed of maxillary and mandibular jaws mounted on an articulator. Each model was covered with removable gingiva and 32 anatomically-shaped teeth, and each jaw included six teeth on which a student could perform extraction practice. Anatomically-shaped extraction teeth can be held in the socket by hot melt (heated glue) with the proper resistance, allowing for repeated extraction training. The TEM jaws were removed from the articulator and then fixed to vertical adjustment in the bottom and upper plates of a mannequin head (*P* − 6/3, Frasaco GmbH, Tettnang, Germany) with a face mask (*P* − 6 G; Frasaco GmbH, Tettnang, Germany) (Fig. 1). The other instrumentation consisted of a universal forceps set (Hu-Friedy, Chicago, IL, USA) and a 3 mm straight luxating Bein elevator (Hu-Friedy, Chicago, IL, USA) for maxillary and mandibular tooth extraction. Preclinical exercises with the TEMs and patient care was performed in the university teaching hospital, participation of the students in the survey took place in the lecture hall of the same university.

### Survey items

In line with the ethical committee’s regulatory requirement, an informed consent form providing sufficient information in an understandable format was embedded in the anonymous survey. Students were informed that participation was voluntary, and their responses would be anonymous and only used for the research purposes of the study aim. Students were not forced to participate in this study, and their decision in volunteering to participate had no effect on their academic standing.

The survey was composed of four main sections. The first section covered two basic questions such as sex (Item 1) and preferences of supplementary materials (handouts, social media, and textbooks) (Item 2). In the second section, four questions sought participants’ perceptions of the preclinical training methods (Items 3 and 4), their self- confidence (Item 5), and their anxiety level (Item 6) prior to performing tooth extraction. Items 3 and 4 were rated with 7- point Likert scales, for which a score of 1 represented “strongly disagree” and a score of 7 represented “strongly agree”. Similarly, Item 5 sought to measure self-confidence levels on a 7- point Likert scale ranging from 1 (not at all confident) to 7 (extremely confident). Students’ anxiety levels were measured with the Interval Scale of Anxiety Response (ISAR) in Item 6. The third section comprised six questions (Items 7 to 12) to determine the students’ competency in various steps of routine tooth extraction technique following the first clinical experience. All questions in this section were rated with 7- point Likert scales ranging from “strongly disagree” to “strongly agree”. In the final section, one question (Item 13) provided a thematic analysis with some free space for additional notes, aiming at obtaining feedback on dental students’ opinions related to all facets of tooth extraction education. The thematic analysis of free-text comments made by the cohorts revealed several comments falling into four themes: challenges in the transition period from preclinical to clinical practice, self-confidence, anxiety levels, and perceptions of surveillance of educators’ supervision.

### Sample size

Before launching the study, the adequate sample size for meaningful comparisons was determined by using the Gpower 3.1 software. The effect size was calculated as 0.30 by relying on Okubo et al.’s [9] study. After that, considering the power of the study as 80% (minimum) and its confidence interval as 95%, it was decided that at least 108 students (54 in each cohort) would be needed.

### Statistical analysis

The data was obtained randomly by using face-to-face group meetings in the university lecture theatre. Then, a blinded investigator (S.S) computed the students’ answers to a Microsoft Excel spreadsheet. The statistical analysis was executed using SPSS software, version 22 (IBM SPSS Inc., Chicago, IL, USA) and data distribution was examined using the Shapiro-Wilk test. In addition to descriptive statistics, statistical and thematic analyses were conducted according to survey responses. Student characteristics were summarized using means and standard deviations and compared for the two cohorts using the t-test. Students’ self-reported anxiety and self-confidence levels were analyzed with this test as well. For all statistical analyses, the significance level was set at *P* ≤ 0.05.

## Results

One hundred and nineteen surveys were filled out by the undergraduate dentistry students (*n* = 136), which correlated to a response rate of 85.2%. A total of 116 qualified surveys were received out of 119; the efficiency was 97.4%. Out of the 116 surveys included in this study, half were found to belong to dental students in Cohort 1 who had the opportunity to train on TEMs. As the proportion of females was 53.2% in Cohort 1 and 58.6% in Cohort 2, the participants appeared to be distributed almost evenly by sex in both cohorts.

Table 1 presents an overview of the supplementary materials utilized by students who endeavored to complement their learning in tooth extraction. While both cohorts preferred social media (e,g. YouTube) and handouts to textbooks for learning purposes, Cohort 2 still showed a significantly higher preference for textbooks compared with Cohort 1 (*P* = 0.04).

**Table 1:** Used supplementary materials. Data are expressed as mean scores ± SD

Item 2: What is your favorite preferred supplementary materials?	Cohort 1	Cohort 2	P-value
Textbooks	2.21 ± 1.3	2.67 ± 1.2	0.040*
Handouts	3.78 ± 1.0	3.86 ± 1.0	0.650
Social media (Youtube etc.)	3.76 ± 1.3	3.88 ± 1.4	0.624

Note SD, standard deviation. (*P* < 0.05; t-test)

The frequency distribution of the responses with 7- point Likert scales is shown in Table 2. The majority of the students in both Cohort 1 and Cohort 2 considered their learning experience to be sufficient for performing their first tooth extraction practice on a real patient, with rates varying between 5 and 7. There were no significant differences in students’ satisfaction level with the learning experience between the cohort groups; however, the students in Cohort 1 reported that they felt significantly more prepared for their first clinical tooth extraction experience (Item 4) than those in Cohort 2 (*P* = 0.028) (Table 3).

**Table 2:** Dental students’ responses for questions regarding their perceptions of the relevant preclinical training methods

	Strongly disagree	Disagree	Somewhat disagree	Neither agree nor disagree	Somewhat agree	Agree	Strongly agree
Item 3: The preclinical training method that I received for my first tooth extraction practice on a real patient was adequately sufficient							
Cohort 1	6 (10.3)	6 (10.3)	3 (5.2)	4 (6.9)	13 (22.4)	15 (25.9)	11 (19)
Cohort 2	2 (3.4)	8 (13.8)	8 (13.8)	11 (19)	15 (25.9)	8 (13.8)	6 (10.3)
Item 4: I feel ready for my first tooth extraction practice on a real patient.							
Cohort 1	2 (3.5)	5 (8.6)	3 (5.2)	14 (24.1)	13 (22.4)	12 (20.7)	9 (15.5)
Cohort 2	6 (3.5)	9 (8.6)	6 (5.2)	8 (24.1)	17 (22.4)	7 (20.7)	5 (15.5)
Item 7: I was confident that I was able to choose proper surgical instruments prior to performing tooth extraction.							
Cohort 1	0 (0)	1 (1.7)	5 (8.6)	6 (10.4)	14 (24.1)	17 (29.3)	15 (25.9)
Cohort 2	1 (1.7)	0 (0)	4 (6.9)	15 (25.9)	18 (31)	12 (20.7)	8 (13.8)
Item 8: I easily positioned my opposite supporting hand to support the jaw and stabilize it during extraction.							
Cohort 1	1 (1.7)	6 (10.4)	3 (5.2)	5 (8.6)	20 (34.5)	16 (27.6)	7 (12)
Cohort 2	2 (3.5)	2 (3.5)	6 (10.3)	10 (17.2)	19 (32.8)	14 (24.1)	5 (8.6)
Item 9: I was quite comfortable with loosening the soft tissue and subsequently luxation of the tooth with a dental elevator.							
Cohort 1	2 (3.5)	0 (0)	10 (17.2)	7 (12.1)	16 (27.6)	14 (24.1)	9 (15.5)
Cohort 2	6 (10.4)	7 (12)	2 (3.5)	8 (13.8)	14 (24.1)	16 (27.6)	5 (8.6)
Item 10: I easily seated the forceps beaks as far as apically and close-fitting position to the tooth root underneath the loosened soft tissue.							
Cohort 1	2 (3.5)	0 (0)	4 (6.9)	3 (5.2)	22 (37.9)	18 (31)	9 (15.5)
Cohort 2	2 (3.5)	2 (3.5)	5 (8.6)	10 (17.2)	17 (29.3)	16 (27.5)	6 (10.4)
Item 11: I was quite capable with the sequential motions performed using the forceps.							
Cohort 1	0 (0)	1 (1.7)	7 (12.1)	7 (12.1)	20 (34.5)	10 (17.2)	13 (22.4)
Cohort 2	2 (3.5)	1 (1.7)	3 (5.2)	18 (31)	19 (32.7)	13 (22.4)	2 (3.5)
Item 12: I do not need surveillance in my next performance of tooth extraction.							
Cohort 1	1 (1.7)	7 (12.1)	4 (6.9)	13 (22.4)	13 (22.4)	9 (15.5)	11 (19)
Cohort 2	2 (3.5)	3 (5.2)	10 (17.2)	8 (13.8)	4 (6.9)	15 (25.9)	16 (27.5)

Note Data expressed are as n (%)

**Table 3:** Comparison between the groups for item 3,4 and 7–12. Data are expressed as mean scores ± SD

Item Number	Cohort 1	Cohort 2	p-value
Item 3	4.74 ± 2	4.32 ± 1.6	0.221
Item 4	4.77 ± 1.6	4.06 ± 1.8	0.028*
Item 7	5.48 ± 1.3	5.01 ± 1.3	0.054
Item 8	4.94 ± 1.5	4.79 ± 1.4	0.571
Item 9	4.94 ± 1.5	4.46 ± 1.8	0.125
Item 10	5.29 ± 1.3	4.89 ± 1.5	0.126
Item 11	5.2 ± 1.4	4.68 ± 1.2	0.033*
Item 12	4.74 ± 1.7	5.03 ± 1.8	0.365

Note SD, standard deviation. (*P* < 0.05; t-test)

Considering the responses related to ease in the selection of proper surgical instruments such as forceps and elevators (Item 7), over half of the students in Cohort 1 either agreed (29.3%) or strongly agreed (25.9%) that they were able to choose the proper instruments (Table 2). As for Cohort 2, most students indicated that they “somewhat agreed” (31%) or “neither agreed nor disagreed” (25.9%) that they experienced ease in selecting the proper instruments. Only a few students from the cohort pointed out their inability to select the proper instruments. There were no significant differences in awareness of selecting proper surgical instruments between Cohort 1 and Cohort 2 (*P* > 0.05) (Table 3).

Cohort 1 expressed a higher degree of comfort in each of the steps of routine tooth extraction, which were enumerated as follows: positioning the opposite hand (Item 8), luxating the tooth with a dental elevator (Item 9), seating the forceps on the tooth (Item 10), and being capable of/comfortable performing sequential motions using the forceps (Item 11). The difference between Cohort 1 and Cohort 2 was significant only for Item 11 (*P* = 0.033) (Table 3).

Tables 4 and 5 provide our findings on students’ self-confidence and anxiety levels, respectively. Whereas Cohort 1 tended to exhibit higher self-confidence and lower anxiety levels than Cohort 2, female students presented more of a lack of self-confidence and higher anxiety. However, there were no significant differences detected between the cohorts or sexes with regard to either self- confidence or anxiety levels (*P* > 0.05).

**Table 4:** Students’ self-reported confidence levels after tooth extraction on a real patient. Data are expressed as n (%) or mean scores ± SD

Item 5: Please rate your self- confidence level before performing tooth extraction on a real patient	Cohort 1	Cohort 2	Female	Male
3	0 (0)	0 (0)	0 (0)	0 (0)
4	2 (3.5)	2 (3.5)	2 (3.2)	2 (4.7)
5	2 (3.5)	4 (6.9)	3 (4.8)	3 (7)
6	7 (12)	15 (25.9)	14 (22.6)	8 (18.6)
7	13 (22.4)	14 (24.1)	16 (25.8)	11 (25.6)
8	17 (29.3)	12 (20.7)	16 (25.8)	13 (30.2)
9	11 (19)	6 (10.3)	11 (17.7)	6 (13.9)
10	6 (10.3)	5 (8.6)	4 (6.5)	7 (16.3)
Mean ± SD	7.68 ± 1.45	7.17 ± 1.5	7.36 ± 1.4	7.52 ± 1.6
*P*-value	*p* = 0.062		*p* = 0.579	

Note SD, standard deviation. Confidence levels ranged from 3 = lowest to 10 = highest (*p* < 0.05; t-test)

**Table 5:** Students’ self-reported anxiety level before performing first tooth extraction on a real patient. Data are expressed as n (%) or mean scores ± SD

Item 6: Please indicate the most relevant definition described below considering your anxiety level before performing tooth extraction on a real patient.	Cohort 1	Cohort 2	Female	Male
Calm and relaxed	6 (10.4)	8 (13.8)	7 (10.6)	7 (14)
A little nervous	30 (51.7)	25 (43.1)	33 (50)	22 (44)
Tense and upset	8 (13.8)	7 (12.1)	7 (10.6)	8 (16)
Afraid	4 (6.9)	7 (12.1)	4 (6.1)	7 (14)
Very afraid	1 (1.7)	0 (0)	1 (1.5)	0 (0)
Panicked	8 (13.8)	7 (12)	14 (21.2)	1 (2)
Terrified	1 (1.7)	4 (6.9)	0 (0)	5 (10)
Mean ± SD	2.86 ± 1.6	3.05 ± 1.8	3.01 ± 1.7	2.88 ± 1.7
*P*-value	*P* = 0.555		*P* = 0.677	

Note SD, standard deviation. (*P* < 0.05; t-test)

The thematic analysis of free-text comments is presented in Table 6. Most students reported that they had some difficulties in their clinical practice, as the TEM did not accurately reflect the realistic attachment rigidity between the tooth and the alveolar bone. The most often mentioned unsatisfactory aspects were related to both lack of anatomical knowledge and operator positions (e.g. “I had difficulties with the anatomy”, and “I was confused about the proper operator position and hand maneuvers during tooth extraction” for Cohort 2. These students reported that they were feeling anxious about causing harm to their patient (e.g. “I’d say it is not a fear, per se, but rather a concern about causing harm to the patient. I worry about harming the patient.“).

**Table 6:** Examples of student comments in cohort groups about oral surgery course, by theme and training method

Item 13: Is there anything else you would like to tell us about…	Free- text comments	
Themes	Cohort 1	Cohort 2
Theme 1: Transitioning into clinical practice	“Pre-clinical and theoretical education before the clinicals prevented many problems I could have had otherwise. Repeating my thermotical information on phantom models was productive.” “No matter how much practice we have in the pre-clinic, surgical clinicals is a different arena. I do not think the models repsesent real anatomy.” “The position and attachment of the tooth inside the alveolar pocket were much more difficult to handle than I anticipated.”	“I had difficulties with the anatomy.” “I was confused about the proper operator position and hand maneuver rules during the tooth extraction.” “Any study activity dedicated to this course (for example working on phantoms, observing tooth extractions, practicing on the patients etc.) would help the student to learn more and better.”
Theme 2: Student self-confidence in performing tooth extraction	“I need to spend more time practicing on patients to gain confidence.” “If I feel that the patient trusts me, then I feel more self-confident.”	“Unfortunately, self-confidence is not enough. I am still likely to panic during extractions, I feel like I need to practice more on patients, I do not think that my theoretical knowledge is lacking.”
Theme 3: Student fear in performing tooth extraction	“I have overcome by fear through practicing on phantom models, I feel more comfortable working on the patients.” “I worry that my hands will shake and the patient will see it.”	“If I know what tooth I will be extracting, then I go over the technique of anestesia, proper operator-patient position and proper operator hand position which makes me less anxious.” “I’d say it is not a fear per se, but rather a concern about causing harm to the patient. I worry about harming the patient.”
Theme 4: Surveillance of educators’ supervision	“When the professor stands next to while I work on the patient me, I get much more anxious.”	“Having the professor surveilling and directing the procedure makes both myself and the patient feel less nervous.” “I feel more confident when the professor surveilling me”

## Discussion

A dental student’s attitude towards, demands from, and perception of oral surgery education can be determined effectively by gathering feedback via a survey analysis. Given the importance of student feedback to course improvement and the evaluation of education, a traditional paper-based survey analysis played a crucial role in the study [17]. Through this survey analysis, we demonstrated that students who had a chance to practice with simulation models felt more prepared for real clinical practice and comfortable using surgical instruments. Moreover, there was a significant difference among students in the use of supporting educational material depending on their training method. The students who did not do any training with simulation models felt the need to refer to text-books more than did their counterparts. Although we found several other positive effects of TEM training on practical skills, the perceived self-confidence and anxiety levels when performing tooth extraction on a real patient did not differ significantly between the cohort groups. However, the thematic analysis showed that TEM training worked in the students’ favor.

Over the past decades, dental educators have faced tremendous changes in dental and medical technology, which hold the key to improving modern teaching methods [18]. Considering day-to-day developments in dental technology, dental educators have reached far beyond the traditional approaches as they have integrated different teaching methods into their repertoire. Most recently, these developments include virtual reality which visually mimics the real world [19]. Although virtual reality provides an environment in which students have the chance to train repeatedly, without the direction of one-to-one tutor supervision or wastage of dental consumables, implementing such systems requires a high investment cost [20].

Simulation model learning strategies, as modern teaching methods, have been widely used in dental education, especially in prosthodontics, endodontics, and operative dentistry, though not so much in oral surgery [5–10]. Hence, there is arguably less information about the efficacy of simulation models on preclinical education in this field [21]. However, given that some of the most serious and irreversible complications in dentistry occur in oral surgery, this teaching method might be paramount. Oral surgery, known as an invasive dental discipline, includes both tooth extraction and local anesthesia education in its curriculum to develop the basic skills of undergraduate dental students [7, 14]. Brand et al. [7] reported that most of the dental faculties in Europe teach the theoretical aspects of tooth extraction in the third year, whereas there is more variation in the introduction of practical aspects to dental curricula, ranging from years 2 to 6. In Turkiye, undergraduate dental students take both oral surgery and dental anesthesia courses in the fall and/or spring semesters of their third year, depending on the curriculum of different dentistry faculties [16]. Although students’ perceptions of their oral surgery training have drawn some attention [11, 16] in Turkiye, to our knowledge, no previous study has examined the effect of TEMs on Turkish dental students. In the current study, students’ attitudes, anxiety, and self-confidence levels before their first tooth extraction on a real patient were also investigated.

Our data indicated that undergraduate students who did not train in the simulation laboratory were significantly more active in getting detailed information by reading textbooks than the students motivated by applying their knowledge during practice in the simulation laboratory. This seemed to capture the conditions under which undergraduate students tried to set aside time dedicated to learning by opening up a textbook in a traditional way. Moreover, another notable finding of our study is that the majority of students in both cohorts reported that social media (e.g., YouTube), as a learning tool, shared equal importance with lecture notes. At this point, it is essential to consider how recent studies have revealed that most free online dental information does not meet reliability criteria, and only 5% of this information is provided by academic personnel [22, 23]. As these sources have become supplementary to formal educational material for most and considering the prevalence of centrality of instruction over social media, educators should consider helping their students find credible and contemporary specimens among these sources [24].

The only items that were significantly different in favor of Cohort 1 were the sense of “feeling ready for tooth extraction” and ease with “use of forceps”. In line with our observations, Brand et al. [7] emphasized that various studies [25–27] advocate the teaching of extraction skills with preclinical training models at numerous dental schools in Europe. Redford et al. [28] stated that the provision of preclinical extraction courses is considered to be of utmost importance to the interplay between competency in using forceps and students’ perceptions. In the present study, the students in the TEM trained group were revealed to have noticeable competency in extraction techniques such as rotation and oro-vestibular movement during exodontia.

In our study, sample size estimation was found to be at least 54 subjects for each cohort by power calculation, similar to previous studies [9]. When scrutinizing the studies conducted to evaluate the efficacy of simulation-based educational interventions, we can see that they have involved a limited number of undergraduate students in trained and untrained groups, with samples of 23 to 42 participants being common [21, 29–31]. In line with those studies, the current study found that despite the lack of significant differences between the students who did and did not receive TEM training, TEMs overall received more positive feedback. Still, as the present study, even with a larger sample size, could not definitively prove the efficacy of TEMs, subsequent studies, or at least citywide survey analyses, investigating the effect of TEMs on students’ perceptions should be conducted.

Any attempt to improve oral surgery education must also focus on the possible effect of sex differences on both the anxiety and self-confidence levels of undergraduate dental students, as this can play a crucial role in students’ motivation and determine their learning strategies [32, 33]. Numerous studies on undergraduate students’ anxiety and self-confidence levels have focused on sex differences and consistently found male students to have an advantage over their female counterparts [32, 34–36]. This is congruent with findings of lower confidence in female dental students who participated in a citywide survey analysis [12]. In the same context, Coughlan et al. [37] found strong evidence that female students were almost twice as concerned the male students. Incidentally, as expected, we also found that male students felt more confident and less anxious during tooth removal on a real patient, albeit without a statistically significant difference.

In the current study, thematic analysis revealed that most undergraduate dental students believed that the more training they received on various educational equipment, the more prepared they would be for when they finally saw patients. This supports the idea that a combination of various teaching methods should be used in clinical training [38]. However, a non-negligible number of undergraduate dental students complained about the TEM not closely imitating the sensation of tooth movement within the alveolar bone during tooth extraction and not adequately providing realistic preparation for real clinical situations encountered in everyday practice. Stelzle et al. [14] also observed that most students put great stock into tooth extraction training on mannequin models. Similarly, the authors reported that the connection between theory and dental practice was rated lower than the overall course, a result ascribed to simulation-based tooth extraction models’ imperfect imitation of real-life conditions. Furthermore, Hanisch et al. [39] notably produced individual 3D-printed models for training on root tip resections, offering more realistic simulations as an alternative to industrially manufactured typodont models.

A move towards a more incorporation of technological equipment into modern education rather than traditional dental education methods would improve such education. If using phantom models in combination with any kind of technological equipment to train dental students might be more beneficial than previously realized, as this study seems to suggest, then educators should lead the way in technology-enhanced education as an opportunity to better prepare undergraduate students for their future profession.

We should agree that this study is not without its limitations. First of all, although Cohort 1, which received tooth extraction training using simulation models, had higher satisfaction and self-confidence levels, and lower anxiety levels, than Cohort 2 did, there were no significant disparities between the groups on those pertinent levels. The aforementioned small sample size problem might account for this phenomenon, but probably not exclusively. Second, in this study, we did not evaluate the undergraduate dental students using an assessment method to compare the competency of trained and untrained groups. Nonetheless, patient feedback could also have been collected to prove the students’ enthusiasm related to TEMs. However, in this study, we specifically focused on putting forward the perception of undergraduate students, at least from a practical standpoint, with the feedback received. Another limitation is that the survey used was created by the authors themselves and was not used before. Although the questions utilized in the survey were influenced by previous studies in some capacity, the novel survey used in this study has not been analyzed for validity and reliability.

The results of this study suggested simulation models for tooth extractions to be a supportive tool and emphasized their prospective importance in oral surgery education. Having practiced tooth extraction on simulation models, students felt more confident and ready for various aspects of their first clinical experience in oral surgery, whether it be selecting the proper instruments or deciding the steps necessary for extraction. Further studies are required to adapt simulation model training into the preclinical courses of oral surgery.

### Electronic supplementary material

Below is the link to the electronic supplementary material.

Supplementary Material 1

## Data Availability

The datasets used and analyzed during the current study are available from the corresponding author upon reasonable request.
