# Supplementary material for: Evaluation of the efficacy of a simulation model used in oral and maxillofacial surgery education
Source: BMC Med Educ. 2024 Mar 19;24:310. doi: 10.1186/s12909-024-05307-3 (PMC10953247; doi:10.1186/s12909-024-05307-3)
Supplement: Supplementary file 1 — Supplementary Material 1 [file 12909_2024_5307_MOESM1_ESM.pdf]

INFORM CONSENT FORM

Dear Students,

Our research, entitled “Evaluation of the effectiveness of surgical tooth extraction simulation models" is led by Dr. Aysegul Erten Taysi (principal investigator) from Altınbas University Faculty of Dentistry, Department of Oral and Maxillofacial Surgery.

This study aims to improve the quality of our educational practices and analyze them comprehensively. We assure you that the information provided by you will be kept confidential and will be used solely for academic purpose.

If you wish to participate, please check the box below:

o I agree to participate in the survey
o I do not agree to participate in the survey

Thank you for your valuable contribution to our study.

**1. General questions**
Item 1. Sex<br>o Woman<br>o Man<br><br>Item 2. What is your favorite preferred supplementary materials? Please give them a score between 1 and 5 (1=definitely not beneficial, 5=definitely beneficial)<br>Textbooks = …..<br>Handouts = …..<br>Social media = …..
**2. The students' perceptions of the preclinical training methods**
Item 3 The preclinical training method that I received for my first tooth extraction practice on a real patient was adequately sufficient<br>o Strongly disagree<br>o Disagree<br>o Somewhat disagree<br>o Neither agree nor disagree<br>o Somewhat agree<br>o Agree<br>o Strongly agree<br><br>Item 4: I feel ready for my first tooth extraction practice on a real patient.<br>o Strongly disagree<br>o Disagree<br>o Somewhat disagree<br>o Neither agree nor disagree<br>o Somewhat agree<br>o Agree<br>o Strongly agree<br><br>Item 5: Please rate your self- confidence level before performing tooth extraction on a real patient.<br>o 1 (not at all confident)<br>o 2<br>o 3<br>o 4<br>o 5<br>o 6<br>o 7 (extremely confident)

Item 6: Please define your anxiety level before performing tooth extraction on a real patient.

- Terrified
- Panicked
- Very afraid
- Afraid
- Tense and upset
- A little nervous
- Calm and relaxed

3. The students' competency in various steps of routine tooth extraction technique

Item 7: I was confident that I was able to choose proper surgical instruments prior to performing tooth extraction

- Strongly disagree
- Disagree
- Somewhat disagree
- Neither agree nor disagree
- Somewhat agree
- Agree
- Strongly agree

Item 8: I easily positioned my opposite supporting hand to support the jaw and stabilize it during extraction.

- Strongly disagree
- Disagree
- Somewhat disagree
- Neither agree nor disagree
- Somewhat agree
- Agree
- Strongly agree

Item 9: I was quite comfortable with loosening the soft tissue and subsequently luxation of the tooth with a dental elevator.

- Strongly disagree
- Disagree
- Somewhat disagree
- Neither agree nor disagree
- Somewhat agree
- Agree
- Strongly agree

Item 10: I easily seated the forceps beaks as far as apically and close-fitting position to the tooth root underneath the loosened soft tissue.

- Strongly disagree
- Disagree
- Somewhat disagree
- Neither agree nor disagree
- Somewhat agree
- Agree
- Strongly agree

Item 11: I was quite capable with the sequential motions performed using the forceps.<br>o Strongly disagree<br>o Disagree<br>o Somewhat disagree<br>o Neither agree nor disagree<br>o Somewhat agree<br>o Agree<br>o Strongly agree<br><br>Item 12: I do not need surveillance in my next performance of tooth extraction.<br>o Strongly disagree<br>o Disagree<br>o Somewhat disagree<br>o Neither agree nor disagree<br>o Somewhat agree<br>o Agree<br>o Strongly agree
**4. A thematic analysis with free text**
Item 13: Is there anything else you would like to tell us about…<br><br>Transitioning into clinical practice:<br><br>Your self-confidence in performing tooth extraction:<br><br>Fear in performing tooth extraction:<br><br>Surveillance of educators' supervision:
